# A scoping review of severe maternal morbidity: describing risk factors and methodological approaches to inform population-based surveillance

**DOI:** 10.1186/s40748-020-00123-1

**Published:** 2021-01-06

**Authors:** Lisa M. Korst, Kimberly D. Gregory, Lisa A. Nicholas, Samia Saeb, David J. Reynen, Jennifer L. Troyan, Naomi Greene, Moshe Fridman

**Affiliations:** 1 Childbirth Research Associates, LLC, North Hollywood, CA USA; 2 Department of Obstetrics and Gynecology, Cedars-Sinai Medical Center, Burns Allen Research Institute, Los Angeles, CA USA; 3 Department of Obstetrics and Gynecology, David Geffen School of Medicine at UCLA, Los Angeles, CA USA; 4 Department of Community Health Sciences, Fielding School of Public Health at UCLA, Los Angeles, CA USA; 5 Maternal, Child and Adolescent Health Division, California Department of Public Health, Sacramento, CA USA; 6 AMF Consulting, Los Angeles, CA USA

**Keywords:** Severe maternal morbidity, Maternal care, Obstetrics, Blood transfusion, Disparities, Quality indicators

## Abstract

**Background:**

Current interest in using severe maternal morbidity (SMM) as a quality indicator for maternal healthcare will require the development of a standardized method for estimating hospital or regional SMM rates that includes adjustment and/or stratification for risk factors.

**Objective:**

To perform a scoping review to identify methodological considerations and potential covariates for risk adjustment for delivery-associated SMM.

**Search methods:**

Following the guidelines for Preferred Reporting Items for Systematic Reviews and Meta-analyses Extension for Scoping Reviews, systematic searches were conducted with the entire PubMed and EMBASE electronic databases to identify publications using the key term “severe maternal morbidity.”

**Selection criteria:**

Included studies required population-based cohort data and testing or adjustment of risk factors for SMM occurring during the delivery admission. Descriptive studies and those using surveillance-based data collection methods were excluded.

**Data collection and analysis:**

Information was extracted into a pre-defined database. Study design and eligibility, overall quality and results, SMM definitions, and patient-, hospital-, and community-level risk factors and their definitions were assessed.

**Main results:**

Eligibility criteria were met by 81 studies. Methodological approaches were heterogeneous and study results could not be combined quantitatively because of wide variability in data sources, study designs, eligibility criteria, definitions of SMM, and risk-factor selection and definitions. Of the 180 potential risk factors identified, 41 were categorized as pre-existing conditions (e.g., chronic hypertension), 22 as obstetrical conditions (e.g., multiple gestation), 22 as intrapartum conditions (e.g., delivery route), 15 as non-clinical variables (e.g., insurance type), 58 as hospital-level variables (e.g., delivery volume), and 22 as community-level variables (e.g., neighborhood poverty).

**Conclusions:**

The development of a risk adjustment strategy that will allow for SMM comparisons across hospitals or regions will require harmonization regarding: a) the standardization of the SMM definition; b) the data sources and population used; and c) the selection and definition of risk factors of interest.

**Supplementary Information:**

The online version contains supplementary material available at 10.1186/s40748-020-00123-1.

## Introduction

The tracking of severe maternal morbidity (SMM) has continued to evolve since it was first initiated by the World Health Organization (WHO) in 2004 as an alternative to maternal mortality surveillance for identifying failures and priorities in maternal health care [1]. By 2009, the WHO adopted a definition for a maternal near-miss (i.e., “a woman who nearly died but survived a complication that occurred during pregnancy, childbirth or within 42 days of termination of pregnancy”) and presented a list of identification criteria [2]. Using this approach, cases are first identified as having “potentially life-threatening conditions” associated with organ system dysfunction or failure. Surveillance relies on medical record review to document clinical, laboratory-based, or management-based SMM criteria. Case identification can occur retrospectively by identifying those who met criteria or prospectively by using a list of potentially life-threatening conditions.

A population-based approach to tracking SMM began in parallel to the WHO approach in 2005, when Wen et al. proposed a definition of SMM using population-based Canadian administrative data [3], which, although less specific compared to medical record data, was more feasible for routine monitoring. In 2008, Callaghan et al. at the United States (US) Centers for Disease Control and Prevention (CDC) published a definition using 15 conditions [4]. The CDC definition was expanded to 25 conditions in 2012 [5], and in 2015 when International Classification of Diseases, Clinical Modification, Version 9 (ICD-9) coding was upgraded to ICD-10, the SMM definition was reduced and consolidated to 21 and then to 18 conditions [6]. Roberts et al. in Australia contributed substantially to these efforts [7], and this work was further adapted in Canada by Joseph et al. [8]. Canadian and Australian SMM definitions were developed in ICD-10.

In the US, in addition to the CDC calculations of national, population-based trends for SMM using administrative data, facility-based SMM case audit (here referred to as “facility-based surveillance”) has been encouraged by both the CDC and the American College of Obstetricians and Gynecologists [9, 10]. In a recent review, Kuklina and Goodman promoted these complementary approaches, asserting that while case audits can go into depth to identify the causes of SMM and suggest avenues for prevention, population-based administrative data can be used not only to examine trends, but also to compare “hospitals, cities, or states” and to develop priorities for research and practice [11]. With funding from the Centers for Medicare and Medicaid services, the National Quality Forum, which provides standards for healthcare quality measurement in the US, has begun to explore the use of maternal morbidity and mortality measures to improve outcomes [12].

To make comparisons (e.g., by region or hospital) interpretable and amenable to policy directives and interventions, it will be necessary to develop a standardized method for adjusting for the most relevant risk factors. To address the research question of how an SMM measure might be adjusted for such comparisons, the objective of this scoping review was to describe what is currently known regarding the risk factors and methodological approaches for studying SMM using routinely collected population-based data.

## Materials and methods

### Data sources and search strategy

We performed a scoping literature review for predictors of delivery-admission SMM, using the definition provided by Anderson et al. [13]: “Scoping studies are concerned with contextualizing knowledge in terms of identifying the current state of understanding; identifying the sorts of things we know and do not know; and then setting this within policy and practice contexts.” This review conformed to guidelines for Preferred Reporting Items for Systematic Reviews and Meta-analyses Extension for Scoping Reviews (PRISMA-ScR) [14]. The PRISMA-ScR checklist is available in Additional file 1. Using the search term “severe maternal morbidity,” the search was conducted in PubMed and Embase, (both initiated in the late 1940’s), and included the entire databases through June 9, 2019. Since SMM is a composite measure that may include as few as one (e.g., intensive care unit admission) or more than 25 indicators, we used only this term (SMM) to narrow our search and retrieve articles using SMM definitions that were most relevant to population-based SMM tracking.

### Inclusion and exclusion criteria

The inclusion criteria were that each study examined SMM as an outcome occurring during the delivery admission and that SMM was presented with risk factor adjustment or stratification. The exclusion criteria were the following: 1) case reports; 2) reviews, letters, or editorials; 3) errata; 4) methodological studies; 5) protocol only studies; 6) descriptive only studies; 7) quality improvement studies; 8) limited outcome studies (i.e., those that did not include a comprehensive SMM composite); 9) studies using SMM as a risk factor/predictor for further complications; 10) qualitative studies; 11) readmission SMM studies; 12) studies focused on SMM due to gynecological conditions (e.g., ectopic pregnancy or spontaneous abortion); 13) studies that relied predominantly on risk factors that were not routinely available in administrative data (e.g., clinical data, laboratory test results); 14) studies that relied on surveillance-based data collection methods (e.g., WHO-based methods); and 15) studies that were not in English. Citations identified through the searches were assessed by one reviewer and verified by another based on title and abstract using these pre-defined criteria. Duplicates were removed. Full text publications of potentially relevant citations were then examined by the same reviewers to assure that eligibility criteria were met.

### Data extraction

Reviewer disagreements about study selection in the full text review and extraction phases were resolved by jointly re-examining studies and reaching mutual agreement. Information regarding each study was then extracted into a pre-defined database. Variables extracted included: study design and eligibility criteria; datasets used; SMM definition used; and risk factors used, including all patient-, hospital-, and community-level risk factors as reported.

### Quality assessment

Risk of bias assessment is not a mandatory part of this review; however, for informative purposes, risk of bias was assessed using a version of the Newcastle-Ottawa Scale (NOS) [15] that was modified to evaluate observational cross-sectional studies relevant to the research question (Additional file 2). Risk of bias was assessed for a single outcome (SMM) within a study. Two reviewers assessed the articles for NOS criteria. Any discrepancy in scoring was resolved by joint re-examination to arrive at consensus. NOS scores were subdivided into those indicating high- (7–10), moderate- (5–6), and low-quality (1–4) of the publication with respect to the research question. Quality scores were used as part of the general assessment of the literature and did not affect the synthesis of results.

### Synthesis of results

Risk factors were categorized into patient-, hospital-, and community-level groups. The patient-level risk factors were classified as pre-existing conditions (e.g., medical or behavioral risk factors), obstetrical conditions (e.g., multiple gestation), and non-clinical conditions (e.g., insurance type). The obstetrical conditions were further divided into those known to exist in the antepartum period (e.g., prior cesarean birth) versus those occurring in the intrapartum or postpartum periods of the delivery admission (e.g., dystocia, delivery mode).

The heterogeneity of the studies included in this review precluded any quantitative synthesis of the effect sizes (odds ratios or relative risks) of the identified risk factors. However, we present a comprehensive list of the risk factors for SMM that were reported in these publications and summarize: 1) the number of studies that used each risk factor; 2) the number of studies for which the effect size for a risk factor was reported to be statistically significant; and 3) the number of studies that did not report an effect size for the risk factor but did one of the following: a) included it in a risk adjustment model, b) stratified analyses by the risk factor, or c) excluded subsets of the population based on the risk factor. This information is presented with the understanding that it was not meaningful to compare or aggregate effect sizes across studies because of the wide heterogeneity of the study designs and covariates in the models.

The synthesis of results was organized by placing the 81 studies in the following categories defined by study approach: 1) *Testing of multiple conditions for association with SMM* without invoking a specific hypothesis; 2) *Hypothesis testing of specific patient-level risk factors*; 3) *Hypothesis testing of hospital-level risk factors*; and 4) *Risk-adjusted SMM rates and trends.* For purposes of this review, both maternal age and race/ethnicity were treated as pre-existing clinical risk factors.

## Results

### Study selection and characteristics

**Fig. 1:**
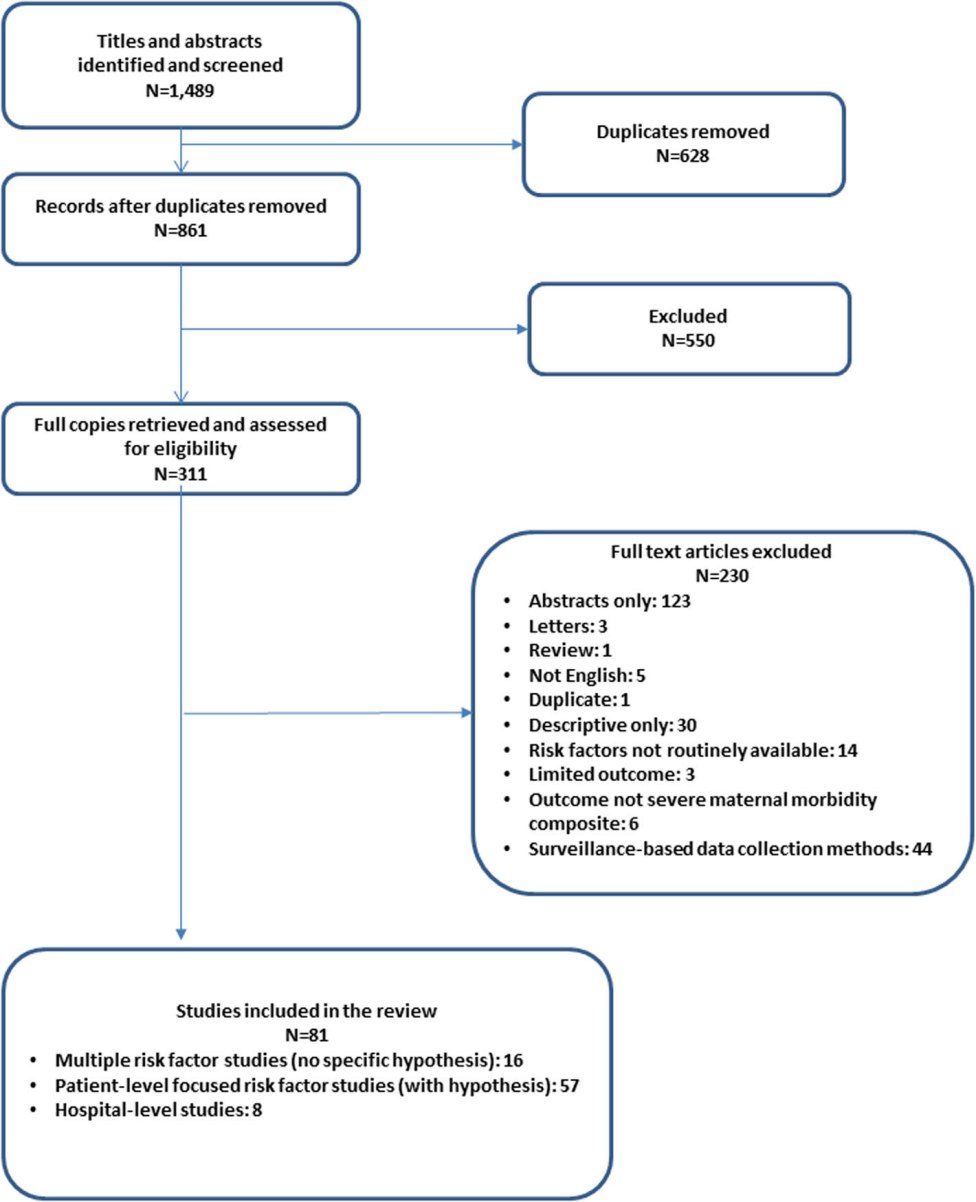
Number of included publications by scoping review step

Results of the literature search are described in Fig. 1. A total of 1489 publications were identified by the original searches, and, of 861 unique titles, 81 (9.4%) met all inclusion and exclusion criteria after thorough review of the full text [3, 8, 16–94]. Table 1 summarizes selected study characteristics and Table 2 presents the risk factors used in the models. Most of the studies (*n* = 71, 87.7%) relied on hospital claims data, medical records, or discharge/birth certificate data from US hospitals. Although some studies sought to examine and identify general clinical risk factors as defined above (*n* = 16, 19.8%), most (*n* = 57, 70.4%) hypothesized an association of SMM with a specific risk factor, such as maternal age or race/ethnicity, or other pre-existing clinical condition; eight studies (9.9%) examined hospital-level risk factors, such as delivery volume. Over one-quarter of the studies (*n* = 22, 27.2%) limited the study population by patient characteristics (e.g., women at low risk) or hospital type (e.g., community hospitals).

**Table 1:** Summary of included studies with reference numbers. *N* = 81 [3, 8, 16–94]

Study Characteristic	Reference Number
**Number and data source of included studies**	
US^a^ data (*n* = 50)	
National Inpatient Sample	17,18,26,39,45,48,58,72
National Readmission Database: 21 states	32
CMS^b^ Medicaid (MAX) data	22
US claims data	27,70
US perinatal data networks	28,43
Hospital discharge data: 7 states	33
Hospital discharge data: Illinois	84
Hospital discharge data: Maryland	81
Hospital discharge ± birth certificate data: New York State	19,44,53
Hospital discharge + birth certificate data: 3 states	29
Hospital discharge + birth certificate data: Iowa	38
Hospital discharge + birth certificate data: New York City	46,47,49,50,78
Hospital discharge, birth, ± death certificate data: California	36,40,52,55-57,68,69,76
Hospital discharge, birth, + death certificate data: Georgia	91
Hospital discharge, birth, ± death certificate data: Washington	42,62–64
Hospital discharge + medical record data: 16 California hospitals	51
Hospital discharge + medical record data: Massachusetts	21
Hospital discharge + SART^c^ + vital statistics data: Massachusetts	23
Birth certificate data: Ohio	71
Birth certificate and SART data: 8 states	67
Medical record data single hospital: California	93
Medical record data single hospital: New York	73
Medical record data single hospital: Missouri	83
Medical record data single hospital: Tennessee	41
Non-US data (*n* = 31)	
Multiple countries including US	60
Multiple countries not including US	90
Japan	16
Korea	75
Sweden	92
Finland	77
France	25,54,88
Canada: Ontario	34,35,80,89
Canada: British Columbia	61,85,86
Canada: multiple provinces	3,8,65,66,74,79,94
Australia: New South Wales	20,24,30,31,37,82,87
Australia: Victoria	59
**Study design and population**	
Study design nested case-control (*n* = 6)	
Yes	16,21,34,42,54,88
Hospital-level risk factors with specific hypotheses (*n* = 8)	
Patient-level data adjusted for clustering among hospitals	19,27,29,32,39
Hierarchical models	36,91
Hospital-level data	49
Delivery discharges limited by: (*n* = 22)	
Community hospitals	36
Annual delivery volume less than 1000	45
Low risk	19,40,65,66
Low risk breech presentation	24
Medicaid eligibility	22
Nulliparous	85
Prior cesarean delivery with parity = 1	94
Prior cesarean delivery ≥3	73
No prior cesarean delivery	66
Cesarean delivery 22–34 gestational weeks	25
Elective cesarean delivery	16
Preterm gestation	20,68
Term laboring patients with prolonged second stage	74
Preeclampsia	27
Hemorrhage	80
Race/ethnicity Hispanic or White	46
Race Black or White	47,48
SMM^d^definition CDC^e^ basis [7] (*n* = 37)	
25 indicators	17,18,23,29,33,36,38,42,46-53,58,60,68,69,71,72,75,76,91,93
18 indicators	19,26,27,41,44,55-57,78,83,84
SMM definition not using CDC basis (*n* = 44)	
Adapted from other US work (Bateman et al. [22])	32,39,45,61–63
Adapted from Canadian work (Wen et al. [3]/Joseph et al. [8])	3,8,22,34,35,66,74,89,94
Adapted from Australian work (Roberts et al. [8])	20,24,30,31,37,59,79,82,87
Adapted from Swedish work (Wahlberg et al. [92])	90,92
Adapted from Geller et al. (5 factor) [95]	43,85
Death	80
Intensive care unit admission	21,25,28,40,53,65,67,71,73
Other	16,54,64,77,86,88
Sensitivity analysis for use of transfusion in SMM definition (*n* = 15)	
Yes	26,33,38,44,46,47,49,50,55-57,60,78,91,94
Blood transfusion threshold was 4 units of packed red blood cells (*n* = 6)	
Yes	43,51,54,85,88,93
Blood transfusion not included in definition (*n* = 10)	
Yes	22,32,37,39,45,77,80,86,90,92
**Key risk factors studied**	
General risk factors (no hypotheses) (*n* = 16)	
Yes	3,8,22,38,42-45,52,53,59,60,80,81,83,90
Patient-level risk factors with specific hypotheses (*n* = 57)	
Race/ethnicity	18,26,33,46-48,50,57,58,72
BMI^f^ or gestational weight gain	35,55,62,71,73,78,86,88
Maternal age	63,76,85
Preterm birth	25,51,68
Infertility/IVF^g^/ART^h^	23,34,54,67,70,93
Preeclampsia	64
Hemorrhage	37
Obstructive sleep apnea	28
Congenital heart disease	79
Inflammatory bowel disease	87
Autoimmunity	30
Idiopathic arthritis	31
Rural vs. urban	61
Maternal birthplace or immigration country	89,92
Amphetamine/opioid use	17
Route of delivery	20,21,24,41,56,66,74,77,84,94
Induction of labor	40,65,82
Anesthesia for cesarean delivery	16
Off-hours delivery	69,75
Hospital-level risk factors with specific hypotheses (*n* = 8)	
Delivery volume	27,29,39
Level of care	32,91
Percent midwives delivering	19
Presence of laborist	36
Hospital quality indicators	49

^a^*US* United States; ^b^*CMS* Centers for Medicare and Medicaid Services; ^c^Society for Assisted Reproductive Technology; ^d^*SMM* severe maternal morbidity; ^e^*CDC* Centers for Disease Control and Prevention; ^f^*BMI* body mass index; ^g^*IVF* in vitro fertilization; ^h^*ART* assisted reproductive technology

**Table 2:** Risk factors tested for association with severe maternal morbidity

Potential covariates for use in risk adjustment	Number of studies that used the variable in risk adjustment	Number of studies with a statistically significant result	Number of studies with a non-significant result	Number of studies where statistical significance was not reported
*Pre-existing clinical*				
Heart disease: including CHF,^a,b^ CHD,^a,c^ pulmonary hypertension,^a^ ischemic heart disease,^a^ valvular heart disease,^a^ conduction disorders	43 (53.1%)	10	0	33
Sickle cell disease^a^	11 (13.6%)	1	0	10
Collagen vascular disease: including SLE^a,d^ and rare autoimmune, rheumatoid arthritis and other collagen vascular	27 (33.3%)	5	0	22
HIV^a^	21 (25.9%)	3	0	18
Chronic renal disease^a^	37 (45.7%)	7	0	30
Chronic hypertension^a^	68 (84.0%)	20	0	48
Chronic diabetes^a^	67 (82.7%)	15	1	51
Chronic lung disease, including asthma^a^	31 (38.3%)	5	1	25
Thyroid disease, hypothyroidism	8 (9.9%)	0	0	8
Maternal soft tissue condition: includes other uterine surgery, fibroids, cervical conditions	5 (6.2%)	0	0	5
Pelvis abnormal	1 (1.2%)	0	0	1
Gynecological conditions: e.g., endometriosis, PCOS,^e^ peritoneal adhesions	3 (3.7%)	0	0	3
Skin and subcutaneous tissues	1 (1.2%)	0	0	1
Hospitalization in prior 5 years or during pregnancy	4 (4.9%)	1	0	3
Drug abuse:^a^ including amphetamine, opioid, other; one vs multiple substance, cocaine, combination with alcohol or smoking or mental health conditions	27 (33.3%)	4	1	22
Alcohol abuse^a^	16 (19.8%)	1	1	14
Smoking	34 (42.0%)	6	7	21
Mental health, including depression	16 (19.8%)	4	0	12
Obesity class, BMI,^f^ weight gain during pregnancy	40 (49.4%)	15	5	20
Height	2 (2.5%)	0	0	2
Weight gain	3 (3.7%)	2	1	0
Liver disorders, including failure, hepatitis B or C	11 (13.6%)	2	0	9
Digestive diseases, including IBD^g^	9 (11.1%)	1	2	6
Seizures and other CNS^h^ conditions, e.g., stroke, MS^i^	12 (14.8%)	2	0	10
Blood diseases: including thrombocytopenia, coagulopathy, or anaemia	16 (19.8%)	3	0	13
Fluid electrolyte disorders	2 (2.5%)	0	0	2
Paralysis	2 (2.5%)	0	0	2
Peripheral vascular disorders	4 (4.9%)	0	0	4
Weight loss	2 (2.5%)	0	0	2
Musculoskeletal conditions	5 (6.2%)	0	2	3
Malignancy	4 (4.9%)	0	0	4
History of organ transplant	1 (1.2%)	0	0	1
High risk summary measure	28 (34.6%)	14	0	14
VTE,^j^ anticoagulant use (now or in past)	4 (4.9%)	1	0	3
Hyperlipidemia	1 (1.2%)	0	0	1
Disorders of the adrenal gland	1 (1.2%)	0	0	1
Obstructive sleep apnea	1 (1.2%)	1	0	0
Genital herpes	4 (4.9%)	0	0	4
Cystic fibrosis	2 (2.5%)	0	1	1
Maternal age^a^	79 (97.5%)	31	2	46
Maternal race/ethnicity	44 (54.3%)	24	0	20
***N*** **= 41 pre-existing clinical covariates**				
*Obstetrical Antepartum*				
Parity (nulliparous vs grand multipara) and combinations with prior cesarean birth	48 (59.3%)	16	0	32
Prior cesarean delivery,^a^ number of prior cesareans	48 (59.3%)	13	2	33
Prior preterm birth	4 (4.9%)	0	0	4
Multiple gestation^a^	69 (85.2%)	15	2	52
Preeclampsia:^a^ including severe, mild, gestational, eclampsia	47 (58.0%)	13	1	33
Placental conditions	25 (30.9%)	6	0	26
Gestational diabetes	29 (35.8%)	4	5	20
Assisted conception: invasive vs. non-invasive, IUI,^k^ ovulation induction, IVF, ICSI,^l^ diagnosed infertility, infertility treatment	13 (16.0%)	7	0	6
Neonatal congenital anomalies or cancer	14 (17.3%)	0	0	14
Fetal presentation	14 (17.3%)	2	2	10
LGA^m^ or SGA^n^ fetus	14 (17.3%)	3	1	10
Oligohydramnios/polyhydramnios	6 (7.4%)	1	0	5
Male fetus	5 (6.2%)	0	0	5
First trimester prenatal care, adequate prenatal care	17 (21.0%)	8	0	9
Provider type (at PNC,^o^ delivery)	4 (4.9%)	1	0	2
Isoimmunization	4 (4.9%)	0	0	4
Number of previous livebirths, number of previous miscarriages/previous miscarriage, ectopic, termination	4 (4.9%)	1	0	3
Prior D&C^p^	1 (1.2%)	0	0	1
History of hemorrhage previous pregnancy	2 (2.5%)	2	0	0
History of hypertensive disorder in a previous pregnancy	1 (1.2%)	1	0	0
History of SGA	1 (1.2%)	0	0	1
Group B streptococcus screen positive	1 (1.2%)	0	0	1
***N*** **= 22 obstetrical antepartum covariates**				
*Intrapartum or postpartum*				
Bishop score < 6	1 (1.2%)	1	0	0
Unengaged fetal head	3 (3.7%)	0	0	3
Uterine rupture, prolapsed cord	2 (2.5%)	0	0	3
Hemorrhage	4 (4.9%)	1	0	3
Prior stillbirth or infant death	4 (4.9%)	0	0	4
Stillbirth	9 (11.1%)	0	0	9
Route of delivery: includes labor y/n, operative VD,^q^ cesarean	35 (43.2%)	12	3	20
Maternal indication for cesarean	1 (1.2%)	0	0	1
Cesarean incision type	1 (1.2%)	0	1	0
Induction	12 (14.8%)	5	0	7
Cervical ripening	1 (1.2%)	1	0	0
Epidural use	3 (3.7%)	0	0	3
PROM,^r^ PPROM^s^	8 (9.9%)	0	0	8
Chorioamnionitis or maternal infection	4 (4.9%)	1	0	3
Birth day: weekend, night	6 (7.4%)	3	2	1
Gestational age at delivery, preterm birth, type of preterm birth	33 (40.7%)	6	0	27
Preterm birth spontaneous vs indicated	2 (2.5%)	0	0	2
Labor anomalies: prolonged second stage, oxytocin	5 (6.2%)	1	0	4
General vs neuraxial anesthesia	3 (3.7%)	2	0	1
Perineal trauma	1 (1.2%)	0	0	1
Elective delivery	6 (7.4%)	0	0	6
Fetal distress not in labor (separate from elective)	1 (1.2%)	0	0	1
***N*** **= 22 intrapartum/postpartum covariates**				
*Other patient-level covariates*				
Year of childbirth	29 (35.8%)	13	2	14
Income	2 (2.5%)	1	0	1
Rural	4 (4.9%)	2	0	2
Insurance	39 (48.1%)	16	4	19
Education	21 (25.9%)	7	0	14
SES^t^	5 (6.2%)	1	0	4
Foreign born	16 (19.8%)	7	1	8
Language spoken	1 (1.2%)	0	0	1
Refugee status	1 (1.2%)	0	0	1
Duration of residence	1 (1.2%)	0	0	1
Working	2 (2.5%)	0	2	0
Married	13 (16.0%)	3	2	8
Profession	1 (1.2%)	0	0	1
Home birth	1 (1.2%)	0	0	1
Transfer in from other hospital	3 (3.7%)	0	0	3
***N*** **= 15 other patient-level covariates**				
*Hospital-level covariates*				
Hospital level of maternal care	2 (2.5%)	1	1	0
Hospital size or delivery volume	17 (21.0%)	6	3	8
Hospitalist	1 (1.2%)	0	1	0
Hospital ownership	11 (13.6%)	4	2	5
Hospital teaching	15 (18.5%)	5	2	8
Hospital urban/rural	9 (11.1%)	2	2	5
Hospital percent high-risk	1 (1.2%)	1	0	0
Hospital percent non-White	1 (1.2%)	1	0	0
Hospital black-serving	1 (1.2%)	1	0	0
Hospital percent Medicaid	3 (3.7%)	1	0	2
Hospital coding intensity	1 (1.2%)	1	0	0
Hospital percent midwife births	1 (1.2%)	0	1	0
NICU^u^ level	4 (4.9%)	2	1	1
Hospital cesarean rate general endotracheal anesthesia	1 (1.2%)	0	0	1
Hospital epidural rate	1 (1.2%)	0	0	1
Hospital induction rate	1 (1.2%)	0	0	1
Hospital percent NTSV^v^	2 (2.5%)	0	0	2
Hospital percent early elective deliveries	2 (2.5%)	0	0	2
Hospital Clinical Processes of Care quintiles	1 (1.2%)	0	0	1
Hospital Patient Perspectives of Care quintiles	1 (1.2%)	0	0	1
Hospital number triaged per day	1 (1.2%)	0	0	1
Hospital number triaged per delivery	1 (1.2%)	0	0	1
Hospital > 4 hospitals within 20 miles of residence	1 (1.2%)	0	0	1
Hospital excellent doctor:nurse relationship	1 (1.2%)	0	0	1
Hospital doctors/1000 deliveries	1 (1.2%)	0	0	1
Hospital MFM^w^ on staff	1 (1.2%)	0	0	1
Hospital midwives available	1 (1.2%)	0	0	1
Hospital anesthesia available 24/7	1 (1.2%)	0	0	1
Hospital anesthesia staff have no other responsibilities	1 (1.2%)	0	0	1
Hospital equivalent staffing day and night	1 (1.2%)	0	0	1
Hospital cesarean in main hospital operating room	1 (1.2%)	0	0	1
Hospital radiology available 24/7	1 (1.2%)	0	0	1
Hospital blood bank available24/7	1 (1.2%)	0	0	1
Hospital massive transfusion protocol in place	1 (1.2%)	0	0	1
Hospital pharmacist dedicated to L&D^x^	1 (1.2%)	0	0	1
Hospital Bakri Balloon available	1 (1.2%)	0	0	1
Hospital epidural easy to get	1 (1.2%)	0	0	1
Hospital adult critical care 24/7	1 (1.2%)	0	0	1
Hospital subspecialty intensive care units available	1 (1.2%)	0	0	1
Hospital difficult to get consults	1 (1.2%)	0	0	1
Hospital has NICU	1 (1.2%)	0	0	1
Hospital central FHR^y^ monitoring	1 (1.2%)	0	0	1
Hospital emergency response team available to L&D	1 (1.2%)	0	0	1
Hospital allow TOLAC^z^	1 (1.2%)	0	0	1
Hospital 100% of cesareans begun within 30 min	1 (1.2%)	0	0	1
Hospital intermittent FHR monitoring < 50% of patients	1 (1.2%)	0	0	1
Hospital doctors sign out to each other	1 (1.2%)	0	0	1
Hospital formal rounds are conducted on L&D	1 (1.2%)	0	0	1
Hospital drills and simulations required	1 (1.2%)	0	0	1
Hospital FHR monitoring course required of doctors	1 (1.2%)	0	0	1
Hospital tracking of haemorrhage occurs	1 (1.2%)	0	0	1
Hospital tracking of infection occurs	1 (1.2%)	0	0	1
Hospital tracking of 3rd & 4th degree lacerations occurs	1 (1.2%)	0	0	1
Hospital has cesarean evaluation team	1 (1.2%)	0	0	1
Hospital allows maternal transfers in	1 (1.2%)	0	0	1
Hospital has a protocol for induction of labor	1 (1.2%)	0	0	1
Hospital has a protocol for cesarean delivery	1 (1.2%)	0	0	1
Hospital gives education regarding induction of labor	1 (1.2%)	0	0	1
***N*** **= 58 hospital-level covariates**				
*Community-level covariates*				
Region	21 (25.9%)	6	1	14
Neighborhood poverty	3 (3.7%)	0	2	1
Miles from zip code to hospital	1 (1.2%)	0	1	0
Geographic designation of area urban/rural	8 (9.9%)	2	0	6
County frequency of obstetricians/anesthesiologists	1 (1.2%)	0	1	0
County frequency of births to teens	1 (1.2%)	0	1	0
County frequency of unmarried women	1 (1.2%)	0	1	0
County frequency of divorced females	1 (1.2%)	0	1	0
County frequency of female family heads	1 (1.2%)	0	1	0
County frequency of females with no insurance	1 (1.2%)	0	1	0
County frequency of foreign-born persons	1 (1.2%)	0	1	0
County frequency of persons with less than high school education	1 (1.2%)	0	1	0
County frequency of non-White persons	1 (1.2%)	0	1	0
County household income measure	18 (22.2%)	4	2	12
County frequency of unemployed persons	1 (1.2%)	0	1	0
County frequency of food stamp beneficiaries	1 (1.2%)	0	1	0
County frequency of persons with no phone	1 (1.2%)	0	1	0
County frequency of households with > 1 person/room	1 (1.2%)	0	1	0
County number of days with good air	1 (1.2%)	0	1	0
County number of deaths due to AIDS^aa^	1 (1.2%)	0	1	0
County number of deaths due to MVA^bb^	1 (1.2%)	0	1	0
County death suicide	1 (1.2%)	0	1	0
***N*** **= 22 community-level covariates**				
**TOTAL:** ***N*** **= 180 covariates**				

^a^included in Bateman Comorbidity Index; ^b^*CHF* congestive heart failure; ^c^*CHD* congenital heart disease; ^d^*SLE* systemic lupus erythematosus; ^e^*PCOS* polycystic ovary syndrome; ^f^*BMI* body mass index; ^g^*IBD* inflammatory bowel disease; ^h^*CNS* central nervous system; ^i^*MS* multiple sclerosis; ^j^*VTE* venous thromboembolism; ^k^*IUI* intrauterine insemination; ^l^*ICSI* intracytoplasmic sperm injection; ^m^*LGA* large for gestational age; ^n^*SGA* small for gestational age; ^o^*PNC* prenatal care; ^p^*D&C* dilatation and curettage; ^q^*VD* vaginal delivery; ^r^*PROM* premature rupture of membranes; ^s^*PPROM* preterm premature rupture of membranes; ^t^*SES* socioeconomic status; ^u^*NICU* neonatal intensive care unit; ^v^*NTSV* nulliparous term singleton vertex; ^w^*MFM* maternal fetal medicine specialist; ^x^*L&D* labor and delivery area; ^y^*FHR* fetal heart rate; ^z^*TOLAC* trial of labor after cesarean; ^aa^*AIDS* acquired immune deficiency syndrome; ^bb^*MVA* motor vehicle accident

### Risk of bias of included studies

Quality scoring is presented in Table 3. Of 10 potential points per study, the median score was 6 (range 3–10). There were 37 high-quality, 39 moderate-quality, and 5 low-quality studies.

**Table 3:** Quality scoring of included studies (*n* = 81)

REFERENCE NUMBER	LAST NAME OF FIRST AUTHOR	YEAR	SELECTION (MAX 3) ***	RISK FACTORS (MAX 4) ****	OUTCOME (MAX 3) ***	TOTAL SCORE
[16]	ABE	2018	**	**	*	5
[17]	ADMON	2018	***	**	*	6
[18]	ADMON	2018	***	**	**	7
[19]	ATTANASIO	2017	*	**	*	4
[20]	BANNISTER-TYRRELL	2015	**	**	*	5
[21]	BARGER	2013	***	**	*	6
[22]	BATEMAN	2013	**	***	**	7
[23]	BELANOFF	2016	***	**	*	6
[24]	BIN	2016	**	**	*	5
[25]	BLANC	2019	*	***	*	5
[26]	BOOKER	2018	***	***	**	8
[27]	BOOKER	2018	**	**	**	6
[28]	BOURJEILY	2017	**	***	*	6
[29]	BOZZUTO	2019	***	**	*	6
[30]	CHEN	2015	**	***	*	6
[31]	CHEN	2013	**	***	*	6
[32]	CLAPP	2018	***	***	**	8
[33]	CREANGA	2014	***	**	**	7
[34]	DAYAN	2019	**	***	*	6
[35]	DAYAN	2018	*	***	*	5
[36]	FELDMAN	2015	***	***	*	7
[37]	FORD	2015	**	**	**	6
[38]	FREDERIKSEN	2017	***	**	**	7
[39]	FRIEDMAN	2016	***	***	**	8
[40]	GIBBS PICKENS	2018	***	**	*	6
[41]	GRASCH	2017	*	***	*	5
[42]	GRAY	2012	***	***	*	7
[43]	GROBMAN	2014	***	****	***	10
[44]	GUGLIELMINOTTI	2019	***	**	**	7
[45]	HEHIR	2013	**	***	**	7
[46]	HOWELL	2017	**	**	**	6
[47]	HOWELL	2016	**	***	**	7
[48]	HOWELL	2016	***	**	*	6
[49]	HOWELL	2014	**	***	**	7
[50]	HOWLAND	2019	**	***	**	7
[8]	JOSEPH	2010	**	**	*	5
[51]	KILPATRICK	2016	***	**	***	8
[52]	KORST	2014	***	***	*	7
[53]	LAZARIU	2017	***	**	*	6
[54]	LE RAY	2019	**	***	***	8
[55]	LEONARD	2019	***	***	**	8
[56]	LEONARD	2019	***	***	**	8
[57]	LEONARD	2019	***	***	**	8
[58]	LIESE	2019	***	**	**	7
[59]	LINDQUIST	2015	**	***	*	6
[60]	LIPKIND	2019	**	**	**	6
[61]	LISONKOVA	2016	**	**	*	5
[62]	LISONKOVA	2017	***	***	*	7
[63]	LISONKOVA	2017	***	***	*	7
[64]	LISONKOVA	2014	***	***	*	7
[65]	LIU	2013	**	*	*	4
[66]	LIU	2007	**	**	*	5
[67]	LUKE	2019	***	**	*	6
[68]	LYNDON	2019	***	**	*	6
[69]	LYNDON	2015	***	**	*	6
[70]	MARTIN	2016	**	***	*	6
[71]	MASTERS	2018	***	***	*	7
[72]	METCALFE	2018	***	***	*	7
[73]	MOURAD	2014	**	**	*	5
[74]	MURACA	2019	*	*	*	3
[75]	NAM	2019	**	**	*	5
[76]	OSMUNDSON	2016	***	**	*	6
[77]	PALLASMAA	2014	**	**	*	5
[78]	PLATNER	2019	**	***	**	7
[79]	RAMAGE	2019	***	*	*	5
[80]	RAY	2018	**	***	*	6
[81]	REID	2018	**	***	**	7
[82]	ROBERTS	2009	**	**	*	5
[83]	ROSENBLOOM	2017	**	****	*	7
[84]	ROY	2019	***	**	*	6
[85]	SCHUMMERS	2018	**	****	*	7
[86]	SCHUMMERS	2015	**	****	**	8
[87]	SHAND	2016	**	***	*	6
[88]	SIDDIQUI	2019	**	****	**	8
[89]	URQUIA	2017	**	*	*	4
[90]	URQUIA	2015	*	*	**	4
[91]	VANDERLAAN	2019	***	***	**	8
[92]	WAHLBERG	2013	**	***	**	7
[93]	WANG	2016		****	***	7
[3]	WEN	2005	**	***	*	6
[95]	YOUNG	2018	**	* *	*	5

### Synthesis of results by study approach

#### Testing of multiple conditions for association with SMM

The 16 publications in this category are described in Table 2. Four of these publications attempted to describe the accuracy of the models using various statistical techniques [22, 43, 44, 83]. All studies used maternal age and 11 used race/ethnicity. In several studies, maternal age [3, 27, 33, 47, 69, 81, 82] and parity [3, 42, 53, 82] appeared to have a U- or J-shaped relationship with SMM, requiring categorization into three or more groups or appropriate selection of the functional form (e.g., polynomial or logistic) for the association of these covariates with SMM. Two studies used no pre-existing risk factors [3, 90] and one used no obstetrical risk factors [60]. Of the 15 studies that did use obstetrical risk factors, four included intrapartum risk factors [3, 38, 44, 53].

#### Hypothesis testing of specific risk factors for SMM

There were 57 studies in this category, and the key risk factors used in modelling are listed in Table 2. As with the studies that tested multiple conditions, maternal age and race/ethnicity were common covariates, as was body mass index (BMI). Where BMI was treated as an independent risk factor, it appeared to have a U-shape. Patients who were underweight and those who were obese had increased risk [42, 53, 55, 62, 63].

There were 10 publications that focused specifically on race [18, 26, 33, 46–48, 50, 57, 58, 72]. SMM rates of Black women have been found to be higher than those of White women, even among those with no comorbidities. In a study by Admon et al., among women with no physical or behavioral health conditions, the SMM rate of non-Hispanic Black women was nearly twice that of non-Hispanic White women [18]. Among women with two or more chronic health conditions, non-Hispanic Black women again had an SMM rate that was nearly twice the rate of non-Hispanic White women. Viewed another way, over time, Metcalfe et al. examined trends of SMM rates by race/ethnicity and found that adjustment for race did not change the SMM trends for 5-year periods between 1993 and 2012, over and above adjustment for comorbidity [72]. Similarly, Leonard et al., in a California study [26], and Booker et al., in a study of older women [57], examined SMM rates over time and found that all racial groups experienced rising SMM; SMM was strongly affected by the presence of comorbidities; and the SMM increases for Black and White women were proportionate. Furthermore, Howland et al. demonstrated that Black-White disparities persisted in the highest income and educational groups [50]. Taken together, these studies suggest that there is a baseline difference in SMM between Black and White women that has not been explained.

Several studies [23, 34, 54, 67, 70, 93] tested whether infertile women were at increased risk of SMM. All found an increased risk for SMM among those receiving infertility treatments, cautioning that this increased risk may be attributable to multiple gestation; however, one publication found SMM risk to be elevated among singleton gestations [70].

There have been separate approaches to including drug, alcohol, and/or tobacco use as covariates in SMM models using administrative data. Some studies incorporated these conditions within a risk factor category labelled mental health while others treated these as separate risk factors. However, the sensitivity of administrative data for this information has been reported to be low [96, 97].

Fifteen studies examined specific intrapartum risk factors for their contribution to SMM: induction of labor [40, 65, 82], off-hours delivery [69, 75], route of delivery [20, 24, 41, 56, 66, 74, 77, 84, 94], and anesthesia type for cesarean delivery [16]. Others included intrapartum risk factors as covariates in the context of other hypotheses or in trying to explain the variation in SMM [21, 25, 37, 41, 46–48, 61, 63, 67, 76, 79]. Multiple investigators specifically used risk-adjustment models that only included antepartum risk factors for SMM to avoid adjustment for differences in patient management.

#### Hypothesis testing of hospital-level risk factors for SMM

Eight studies focused on hospital-level risk factors [19, 27, 29, 32, 36, 39, 49, 91]. There were few consistent findings. Three studies focused on annual hospital delivery volume and had mixed results [27, 29, 39]. Three other investigations tested various hypotheses regarding an association between the following specific hospital characteristics and SMM and found no association: the use of laborists in community hospitals [36], hospital quality indicators [49], and the percent of practitioners doing deliveries at the hospital that were midwives [19].

Given patients with the same high-risk conditions, it has been assumed that delivery at higher level hospitals will lead to less SMM. However, evidence for this supposition is limited. Two studies attempted to find an association between hospital resources and SMM. In both cases, hospital resource levels were studied as proxies for levels of maternal care, which are proposed designations for hospitals based on their resources and staffing [98]. Vanderlaan et al. used American Hospital Association data indicating the risk level of patients cared for by the hospital [91], and Clapp et al. assigned risk levels to patients based on Bateman’s Obstetrical Comorbidity Index and then rated hospitals as high versus low acuity based on their percentages of high-risk patients [32]. In spite of extensive sensitivity analyses, Vanderlaan et al. found no relationship between these proxy resource levels and SMM [91]. Clapp et al. found that high-risk patients had a higher absolute risk of SMM at low-acuity hospitals when compared with high-risk patients at high-acuity centers; however, 95% confidence intervals overlapped and no *p*-value for the comparison was reported [32].

#### SMM rates and trends

Twenty-seven of the included studies presented SMM rates. Several examined trends of SMM rates over the years [17, 26, 38, 45, 72], reporting rising rates of both SMM and associated comorbidities. Some investigators disaggregated SMM rates and reported rates of the various indicators [18, 89]. SMM rates were highly dependent on the SMM definitions, study populations, and adjustment models. For example, some investigators built on the CDC definitions [52, 61–64]; others used broad definitions that included maternal intensive care unit admission [21, 25, 28, 40, 53, 65, 67, 71, 73]. A number of studies extended SMM case finding to 42 days postpartum or readmission with SMM.

In the last 5 years, and particularly with the use of administrative data wherein the number of units of packed red blood cells cannot be reliably ascertained, investigators have recognized that blood transfusion accounts for a large proportion of the SMM cases, and, consequently, whether or not it is included in the SMM definition substantially affects the SMM rate and its interpretability [7]. Fifteen studies did sensitivity analyses to display trends or determine if the effect sizes of risk factors were confirmed when transfusion was eliminated from the SMM definition. Trends from year to year were less likely to show statistical differences, and most studies (with some exceptions [50, 55, 78, 94] showed minimal to no changes in the magnitude of risk factors when excluding transfusion. Another 10 did not include transfusion in their SMM definition, nine studies using a maternal ICU admission did not separate transfusion out, and six used medical chart review to assure that at least 4 units of packed red blood cells were used to qualify as meeting the SMM definition (Table 1).

From the seven studies using administrative data with unrestricted delivery populations and including transfusion in the SMM definition [3, 8, 38, 44, 53, 60, 81], SMM rates varied from 0.44% [3] to 2.55% [53]. Using the US National Inpatient Sample [99], the CDC reported the most recent SMM rates from 2014 as 1.44% with transfusions and 0.35% when using the definition excluding transfusions [7]. The overall rate of SMM increased 200% from 1993 to 2014 when transfusion was included and 20% in the same time period when transfusion was excluded.

Maternal death is not an exclusion criterion for the CDC definition of SMM [7]. Some studies specifically included maternal death whether or not SMM was reported. One posited that the coding of death without SMM must be erroneous, and, therefore, excluded such cases [70]. Friedman et al. studied both SMM and death, finding that: 1) 78.7% of deaths in the dataset had been identified as having SMM (these deaths were referred to as “failure to rescue”); and 2) 1.0% of patients with SMM died [39]. This study did not extend the SMM definition to include post-discharge follow-up. In a study by Ray et al., 68.0% of deaths in a population-based delivery cohort had been identified as having SMM [80].

## Discussion

### Principal findings

This review identified 81 studies of SMM that relied on risk adjustment of routinely collected population-based data. Although the key search term was deliberately chosen to be “severe maternal morbidity” in an attempt to identify studies that incorporated similar outcomes, only 37 (45.7%) used an SMM definition with a CDC basis; the SMM definitions used in the remaining studies varied to a much larger extent. The inclusion of blood transfusions (yes/no) in the SMM definition added a layer of complexity to the comparability of these analyses, given that, in various studies, more than half of the SMM cases had this single indicator of SMM. Such heterogeneity was also evident in the principal datasets used (e.g., claims data, electronic medical record or medical record data, administrative data in both ICD-9 and ICD-10), which may have included linkages to other datasets (e.g., infertility, birth certificate, hospital surveys, census data). Study populations also differed with respect to the definition of a delivery admission and, depending on the purpose of the study, the inclusion and exclusion criteria.

The covariates used for risk adjustment also varied extensively (*n* = 180, Table 2), not only with respect to the choice of covariates, but also with respect to their definitions (e.g., BMI as a continuous, ordinal, or binary variable). Interpretation of the results also depends on the study design (e.g., subset of deliveries included) and model specification (e.g., other covariates included). Some studies attempted to limit the types of covariates to patient-level conditions that would be apparent prior to the childbirth admission, while others attempted to develop more explanatory models for SMM and included intrapartum variables such as dystocia and delivery mode. Several studies used hospital characteristics (e.g., delivery volume, ownership, or teaching status) or community-level variables (e.g., median household income, percent foreign-born by zip code or county) to make comparisons more interpretable or models more explanatory. Consequently, effect sizes (odds ratios and relative risks) could not be synthesized in a meaningful way.

### Interpretation

The call for facility-based surveillance of SMM through case review [10, 11] remains critical for identifying SMM causes, so that prevention strategies and interventions can be developed, implemented, and tested prospectively. In addition, there remains a role for population-based administrative data to describe and monitor the SMM burden [12]. The use of administrative data enables the development of standard SMM rates that can be used to describe trends and disparities and, potentially, to make comparisons across regions and hospitals. Such comparisons can highlight regions or hospitals with disproportionate burdens and can potentially provide insight regarding the quality of pregnancy care for those with SMM and/or the resources needed to address SMM. This use of administrative data at the population level can also inform decisions regarding the potential for public health interventions, such as improving the availability of preconception care [100], and can be used to track their success. Demonstrated success could mean more resources can be deployed to scale-up effective interventions and attenuate the SMM burden.

The results of this review point to several areas that are in need of development for the continued evolution of SMM tracking using population-based data. First is the standardization of the SMM definition. In the US, this definition has been gravitating toward that used by the CDC. However, differences remain across recent US studies, particularly with respect to the inclusion of blood transfusion. The role of transfusions in the administrative definition of SMM needs further evaluation and standardization because the rise in transfusions is due largely to quality improvement efforts to decrease mortality from postpartum hemorrhage [101]. It is apparent that blood products are increasingly being used as part of a secondary prevention effort and that such usage in practice (which is life-saving) conflicts with the interpretation of the SMM measure as a poor outcome.

The second area in need of development is the standardization of the content and size of datasets used for hospital or regional comparisons. Hospital discharge datasets appear to be the best choice because they are relatively similar and nearly universally available. The marginal benefit for the addition of linked patient-level datasets, such as the birth certificate data, may be too resource-intensive for some states. The linkage of a basic subset of hospital variables such as ownership, delivery volume, and teaching status, could be gleaned from a variety of sources and maintained in a central location for consistent use. The importance of community-level variables (e.g., by census tract, zip code, county) has not been well-explored in the literature and needs further evaluation, especially as it relates to the potential for public health intervention and ability to impact SMM rates. Community-level summary measures (e.g., median income, rural status) were frequently used as proxies for patient- or hospital-level comparisons and were relatively infrequently reported as contributing to risk adjustment models.

Third is the selection and definition of risk factors of interest. This will depend on the purpose of the risk adjustment. For the purpose of comparing hospital SMM rates, we suggest that models should adjust for case-mix using the risk factors known upon admission but without including those variables describing intrapartum management (e.g., route of delivery) because these variables are under the control of a given hospital and there is no need to keep them balanced across hospitals. Hospital-level factors, such as resources or staffing characteristics, should also be excluded if hospitals are being ranked. The “within” hospital correlation in SMM can be addressed using clustered standard errors. A more serviceable comparison can be achieved by comparing only hospitals of the same type (e.g., teaching hospitals or community hospitals). By confining hospital comparisons to a group with a similar type, the average hospital for that type yields a better representation of the group compared with an average hospital in a group composed of diverse hospital types. On the other hand, if the purpose is to predict the SMM risk, it is reasonable for these models to include intrapartum-, hospital-, and community-level risk factors to increase explanatory power.

Furthermore, the inclusion of patient-level non-clinical variables (e.g., insurance type, educational level) in SMM risk adjustment models deserves reflection. Such variables may be potential proxies for unmeasured clinical risk factors (e.g., malnutrition), measures of access to higher quality of care, or sources of variation due to discrimination. The risk adjustment purpose and the hypothesized source for the variation in SMM risk due to such variables should determine their use in modelling. For example, for hospital comparisons, use of these covariates would not be appropriate given that they would credit hospitals for poor care given to disadvantaged patients.

### Limitations of the review

As discussed in depth above, a limitation of this review is the study heterogeneity, which prevents meaningful synthesis of effect sizes. More narrow inclusion criteria may have allowed for increased detail regarding the relative importance of specific risk factors, such as race/ethnicity and prematurity.

## Conclusions

This review identified multiple potential risk factors associated with SMM. The heterogeneity of the studies precluded any quantitative synthesis of the effect sizes (odds ratios or relative risks) of the identified risk factors. The development of a risk adjustment strategy that will allow for SMM comparisons across hospitals or regions will require harmonization regarding the standardization of the SMM definition, the datasets and population used, and the selection and definition of risk factors of interest. The ability to perform such comparisons would contribute to the capacity of public health systems to monitor SMM trends and disparities and to develop strategies to decrease the SMM burden. Administrative data comparisons also allow for evaluating the potential for interventions, tracking their success, and estimating the resources needed to scale-up effective interventions.

## Supplementary Information

**Additional file 1.** Newcastle-Ottawa Quality Assessment Scale (adapted for cross sectional studies with healthcare data for research question: What are the risk factors for severe maternal morbidity associated with the delivery admission?

**Additional file 2.** Preferred Reporting Items for Systematic reviews and Meta-Analyses Extension for Scoping Reviews (PRISMA-ScR) Checklist.

## Data Availability

No data or materials were used for the publication of this article beyond the review of those articles referenced.

## Abbreviations

AIDS: Acquired immune deficiency syndrome
ART: Assisted reproductive technology
BMI: Body mass index
CDC: Centers for Disease Control and Prevention
CHF: Congestive heart failure
CHD: Congenital heart disease
CNS: Central nervous system
CMS: Centers for Medicare and Medicaid Services
D&C: Dilatation and curettage
FHR: Fetal heart rate
IBD: Inflammatory bowel disease
ICD-9,10: International Classification of Diseases, Clinical Modification, versions 9,10
ICSI: Intracytoplasmic sperm injection
IUI: Intrauterine insemination
IVF: In vitro fertilization
L&D: Labor and delivery area
LGA: Large for gestational age
MFM: Maternal fetal medicine specialist
MS: Multiple sclerosis
MVA: Motor vehicle accident
NICU: Neonatal intensive care unit
NOS: Newcastle-Ottawa Scale
NTSV: Nulliparous term singleton vertex
PCOS: Polycystic ovary syndrome
PNC: Prenatal care
PRISMA-ScR: Preferred Reporting Items for Systematic Reviews and Meta-analyses extension for scoping reviews
PROM: Premature rupture of membranes
PPROM: Preterm premature rupture of membranes
SART: Society for Assisted Reproductive Technology
SES: Socioeconomic status
SGA: Small for gestational age
SLE: Systemic lupus erythematosus
SMM: Severe maternal morbidity
TOLAC: Trial of labor after cesarean
VD: Vaginal delivery
VTE: Venous thromboembolism
US: United States
WHO: World Health Organization
