# Supplementary material for: A scoping review of severe maternal morbidity: describing risk factors and methodological approaches to inform population-based surveillance
Source: Matern Health Neonatol Perinatol. 2021 Jan 6;7:3. doi: 10.1186/s40748-020-00123-1 (PMC7789633; doi:10.1186/s40748-020-00123-1)
Supplement: Supplementary file 1 — Additional file 1. Newcastle-Ottawa Quality Assessment Scale (adapted for cross sectional studies with healthcare data for research question: What are the risk factors for severe maternal morbidity associated with the delivery admission? [file 40748_2020_123_MOESM1_ESM.pdf]

Additional File 1. Newcastle-Ottawa Quality Assessment Scale (adapted for cross sectional studies with healthcare data for research question: What are the risk factors for severe maternal morbidity associated with the delivery admission?

Selection: Maximum 3 stars

1) Representativeness of the sample:
    a. Truly representative of the average in the US or a US state population. *(all subjects or random sampling)
    b. Somewhat representative of the average in the US or a US state population. * (non-random sampling)
    c. Selected group of users or non-US populations.
    d. No description of sampling strategy
2) Sample size:
    a. Justified and satisfactory. *
    b. Not justified.
3) Inclusion/exclusion criteria:
    a. Comparability between included/excluded/missing subjects or data is established, and the percent of the base population included is satisfactory (>80%).*
    b. The percent of the base population included is unsatisfactory, or the comparability between subjects or data included/excluded is unsatisfactory.
    c. No description of the characteristics of those who were and were not included, or those who did and did not have missing data.

Risk Factors: Maximum 4 stars

1) Ascertainment of the patient-level independent variables (risk factors)
    a. Includes complete or partial review of medical records.**
    b. Based solely on administrative data.*
    c. Other measurement tool.
2) Patient-level confounding factors are controlled.
    a. The study controls for patient-level factors, including demographic factors, and clinical and obstetrical comorbidities (at least one in each category), and is not over-controlled (i.e., does not control for route of delivery or other intrapartum variables; mediation analysis is acceptable)**
    b. The study controls for patient-level factors, including demographic factors, and clinical and obstetrical comorbidities (at least one in each category), and controls for intrapartum variables without using mediation analysis.*
    c. The study does not adequately control for patient-level risk factors.

Outcome: Maximum 3 stars

1) Assessment of the outcome:
    a. Includes complete or partial review of medical records, with an attempt to evaluate or mitigate the impact of transfusion on the outcome.**
    b. Based solely on administrative data, with an attempt to evaluate or mitigate the impact of transfusion on the outcome.*
    c. Either of the above with a severely limited outcome definition (death, antepartum only), or an expanded definition based on intensive care unit admission or including pre-existing conditions, or no attempt to evaluate or mitigate the impact of transfusion on the outcome.
2) Statistical test:
    a. The statistical test used to analyze the data is clearly described and appropriate, and the measurement of the association is presented, including confidence intervals and the probability level (p value).*
    b. The statistical test is not appropriate, nor described, or incomplete.
